# Microtubular TRIM36 E3 Ubiquitin Ligase in Embryonic Development and Spermatogenesis

**DOI:** 10.3390/cells11020246

**Published:** 2022-01-12

**Authors:** Martina Mascaro, Inês Lages, Germana Meroni

**Affiliations:** Department of Life Sciences, University of Trieste, 34127 Trieste, Italy; MARTINA.MASCARO@phd.units.it (M.M.); INES.FERNANDESLAGES@phd.units.it (I.L.)

**Keywords:** tripartite motif (TRIM) E3 ubiquitin ligases, TRIM36, microtubules, embryonic development, spermatogenesis

## Abstract

TRIM36 is a member of the tripartite motif (TRIM) family of RING-containing proteins, also known as Haprin, which was first discovered for its abundance in testis and found to be implicated in the spermatozoa acrosome reaction. TRIM36 is a microtubule-associated E3 ubiquitin ligase that plays a role in cytoskeletal organization, and according to data gathered in different species, coordinates growth speed and stability, acting on the microtubules’ plus end, and impacting on cell cycle progression. TRIM36 is also crucial for early developmental processes, in *Xenopus*, where it is needed for dorso-ventral axis formation, but also in humans as bi-allelic mutations in the TRIM36 gene cause a form of severe neural tube closure defect, called anencephaly. Here, we review TRIM36-related mechanisms implicated in such composite physiological and pathological processes.

## 1. Introduction

The tripartite motif (TRIM) family represents the largest sub-family of RING domain-containing proteins. In addition to an N-terminal RING domain, these proteins share the presence of one or two additional Zn-binding B-box domains (B-box 1 and B-box 2) and a coiled-coil region, hence the term tripartite motif (TRIM) or the acronym RBCC to indicate this module and the entire family [1]. The RING domain confers E3 ubiquitin ligase activity within the ubiquitination cascade to the TRIM family members [2,3]. Additionally, E3 ligase-independent biological roles of TRIM proteins have been proposed, e.g., RNA-binding [4]. As ubiquitination regulates the stability and activity of many proteins, if not all, it is no surprise that this family is implicated in a variety of cellular processes, from transcription to apoptosis, from cell cycle regulation to signal transduction [5,6,7]. This involvement, often associated with spatial and temporal specific expression, implicates TRIM proteins in many physiological processes and pathological conditions. These pathologies are multiple and, frequently, each TRIM family member can have pleiotropic functions. For example, controlling oncogenes and tumor suppressors, several TRIM E3 ligases participate in neoplastic processes, often in a context-dependent manner [5,8]. Another frequent feature within the TRIM family is the implication of many of its members in innate immunity pathways, either as positive or negative regulators of the cellular response to invading pathogens, whether viruses or bacteria, and in autoimmune disorders [9,10].

**Figure 1:**
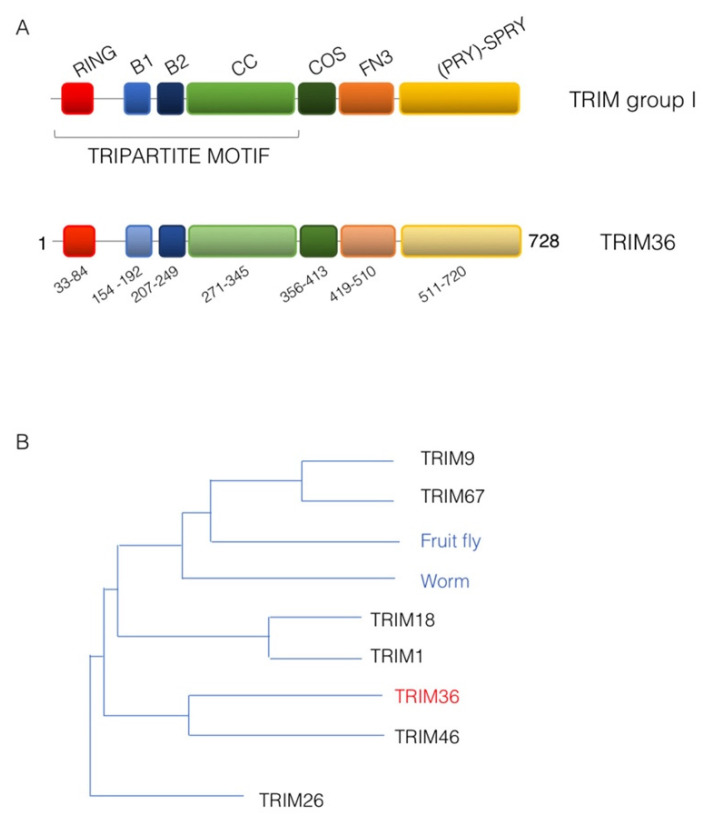
TRIM36 domain structure. (**A**) Schematic representation of the domain structure of TRIM sub-group I family members (upper scheme) and human TRIM36 with the same color code and the indication of the residue limits predicted for each domain as per UniProt Q9NQ86 (www.uniport.org, accessed on 7 December 2021) (lower scheme). RING, RING domain; B1, B-box 1 domain; B2, B-box 2 domain; CC, Coiled-coil region; COS, C-terminal subgroup one signature; FN3, Fibronectin type 3 repeat; SPRY, based on a sequence repeat discovered in the splA kinase and ryanodine receptors. (**B**) Phylogenetic tree showing the evolutionary relationship of TRIM sub-group I family members. The human TRIM family members are shown together with the only member of this sub-group present in invertebrates, represented here by the fruit fly (*Drosophila melanogaster*, Dmel, CG31721) and the worm (*Caenorhabditis elegans*, *madd-2*).

According to the domain(s) displayed C-terminal to the tripartite motif, an extra sub-classification of the TRIM family, counting more than 70 members in humans into at least nine groups, was proposed [11]. Subclass I TRIM family members are characterized by the presence in the tripartite motif of both B-box 1 and B-box 2, following the RING domain and preceding the coiled-coil region. Subclass I feature is a composite C-terminal portion that includes: a COS (C-terminal subgroup one signature) domain, employed by these proteins to associate with the microtubules, followed by a fibronectin type III repeat (FN3) and a PRY-SPRY domain [11] (Figure 1A). The members of this subgroup are indeed all associated with the microtubular apparatus and are mainly expressed during embryonic development where they are often involved in the definition of several developing structures [11,12,13,14]. Accordingly, mutations in some of these genes are associated with paediatric pathologies caused by developmental malformations [14]. 

Here, we will review for the first time the diverse findings reported for TRIM36, a member of TRIM subgroup I, which is implicated in the regulation of spermatogenesis and in early embryonic development.

## 2. Tripartite Motif 36 (TRIM36)

In mammals, TRIM subgroup I is composed of six evolutionarily close members that present orthologous genes in vertebrate species but that are represented only by a single ancestral homologue in invertebrate (Figure 1B) [11,15]. The TRIM36 protein shares the classic TRIM subgroup I domain composition and is 728 amino acids long in humans (UniProt Q9NQ86) (Figure 1A). It was first identified in 2003 in mouse and named Haprin (from the haploid germ cell-specific RBCC protein) in a haploid germ cell specific cDNA library of mouse testis [16]. The human ortholog, *RBCC728/TRIM36*, of the *Haprin* gene was then identified and mapped on chromosome 5q22.3 [17]. From hereafter, we will use the name TRIM36 for this family member.

As for its initial identification, in adult mice, the *Trim36* transcript was confirmed to be abundantly expressed in testis with barely undetectable expression in other organs. Upon testis fractionation, consistent with the origin of its identification, the transcript was detected in the germ cell fraction and was not present in somatic cells, e.g., Sertoli and Leydig cells. Furthermore, the transcript was not found in testis until the fourth postnatal week, when elongated spermatids appeared, to then increase with age [16]. A corresponding trend was observed for its protein product [16]. Expression in testis was confirmed in humans where three transcript isoforms sharing the same expression pattern were reported. Two of these are predicted to produce full TRIM36 proteins displaying few amino acid differences at the very N-terminus before the RING domain, whereas the third isoform is predicted to produce non-functional peptides, if any [18] (UniProt Q9NQ86). During mouse development, *Trim36* was detected by RNA in situ hybridization in the neural tube and developing brain in E14.5 embryos [19]. The *Xenopus trim36* ortholog is also expressed in testis, in addition to the ventral germ plasm region of the oocyte and developing neural tube (see below) [20].

## 3. TRIM36 Associates with the Microtubules

**Figure 2:**
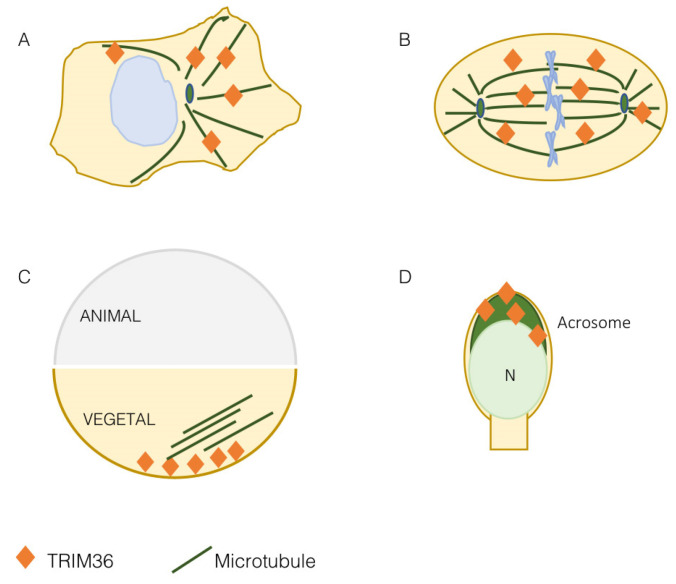
TRIM36 is a microtubule-associated protein. TRIM36 is associated with the microtubular apparatus in mammalian cells both at interphase (**A**) and on the mitotic spindle (**B**). In the *Xenopus laevis* egg, both before and after fertilization, TRIM36 is present in the vegetal pole to organize the microtubule array needed to guide dorsal determinants during cortical rotation (**C**). In elongated spermatids and spermatozoa, TRIM36 is detected in the acrosome (dark green); N, nucleus (**D**).

At first, TRIM36 was found to decorate filaments in the cytoplasm of transfected COS-7 cells and the same was observed for the endogenous protein in the 22RV1 and LNCaP cell lines [17]. This subcellular localization was then confirmed and assessed as microtubular during the identification and characterization of the TRIM group I specific microtubule-interacting domain, the COS (C-terminal subgroup one signature) box in COS-1 cells [11]. This result was confirmed in other cell lines where co-localization with intermediate filaments was indeed excluded and the presence of TRIM36 was also observed on mitotic spindle and cytokinetic midbody microtubules [19,21] (Figure 2A,B). Microtubules are essential protein polymers that serve as structural elements in eukaryotic cells and whose dynamics is tightly controlled for proper cytoskeletal organization. Microtubules have multiple roles within the cell, e.g., capture membrane-bound organelles and remodel membranes, organize the mitotic spindle for correct segregation of chromosomes, cross-link with the actin cytoskeleton and adhesion hubs, define the position of the actomyosin contractile ring during cytokinesis, define cell shape and sense/transduce mechano-tension forces [22].

TRIM36 association with the microtubules underlies a functional effect exerted on this cytoskeletal apparatus. Silencing TRIM36 in HeLa and LN229 cell lines leads to disrupted microtubules and disorganized mitotic spindles [19]. The effect on the spindle can correlate with the observed reduced cell proliferation and increased apoptosis and with the opposite observation when exogenous TRIM36 was expressed [19]. However, this aspect should be further investigated as another report found a markedly decreased cell growth rate following TRIM36 overexpression in NIH3T3 [21]. Whether this inconsistent finding is due to different experimental conditions and cell types or a subtle dosage effect of TRIM36 is an interesting issue to unravel, having a considerable impact also on tumorigenesis as briefly discussed below. Nonetheless, a role in mitosis is supported by the interaction of TRIM36 with CENP-H, which is one of the kinetochore proteins that plays an important role in chromosome segregation [21]. Although the functional role of TRIM36-CENP-H interaction is still unknown, it is interesting that alterations in CENP-H protein levels are associated with the disappearance of the centromere from mitotic chromosomes and, as a consequence, to missing chromosomes during segregation and cell cycle delay [23].

More data on the role of TRIM36 on the microtubular apparatus were gathered when this TRIM was found to be implicated in the organization of the *Xenopus* egg vegetal microtubular array [20] (Figure 2C). As detailed in the next section, in eggs depleted of maternal *trim36*, no vegetal microtubule assembly and cortical rotation are observed. Indeed, *trim36*-depleted eggs either lacked microtubule organization completely or exhibited fragmented microtubules in a loose organization [20]. This effect was rescued by injection of *trim36* mRNA, displaying normal microtubule array formation, but not by a catalytically inactive C217A/H220A *trim36* mutant indicating that the E3 ubiquitin ligase activity is required for vegetal cortical microtubule assembly [20]. Depletion of *trim36* was not affecting microtubule growth and polymerization during the initial phases of cortical rotation, but subsequent cortical displacement did not initiate in *trim36*-depleted embryos and this effect was accompanied by the lack of formation of robust parallel arrays [24]. The precise organization of microtubules makes them polar structures displaying a directionality throughout their length with two different ends. Polarity is essential for many microtubule functions, as it enables directed movements of motor proteins on the microtubule surface, providing tracks for long-distance, motor-driven transport of vesicles and organelles, and for microtubule sliding. Polarity underlies differences in the kinetics of subunit addition and loss at the two microtubule ends. The faster-growing end is called the ‘plus end’ and the slower growing end is referred to as the ‘minus end’ [22]. Plus ends’ detection in *Xenopus* eggs confirmed that, upon *trim36*-depletion, their number was reduced and their behavior chaotic. In addition, tracking of microtubule plus ends growth was non-directional in *trim36*-depleted eggs, which correlates with lack of cortical rotation. This led to conclude that *trim36* might act to coordinate plus end growth thus differentially modulating growth speed and stability of local vegetal arrays necessary for cortical rotation [24].

## 4. TRIM36 Function in Embryonic Development

**Figure 3:**
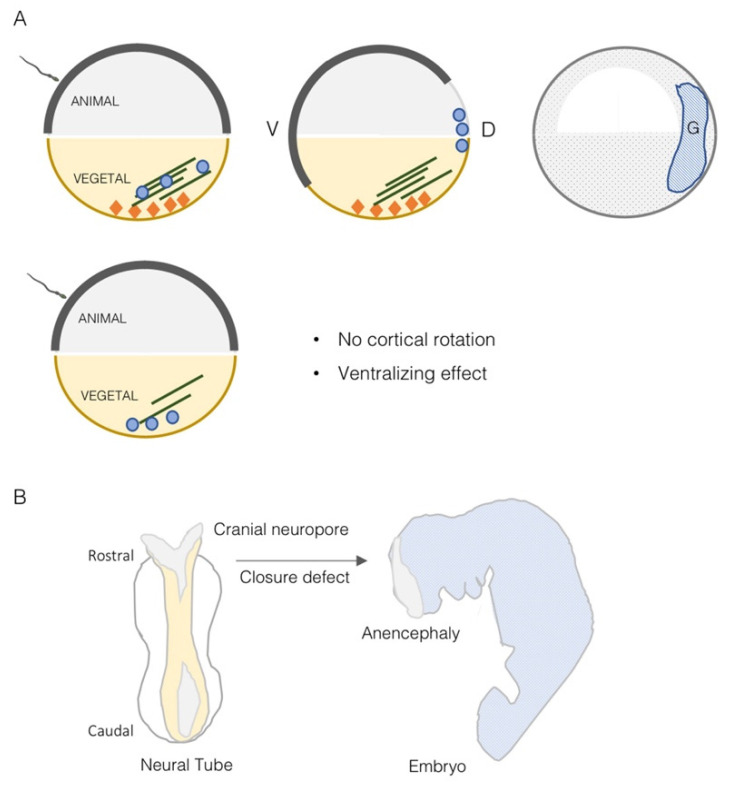
TRIM36 in *Xenopus* early development and in mammalian neural tube closure. (**A**) Scheme showing the normal *trim36* localization (orange diamonds) in the vegetal pole of *Xenopus* oocyte (upper left scheme) and the animal pole cortical rotation, induced after fertilization, which guides dorsal determinants (blue circles) along the microtubular array to define the dorso-ventral axis (D, dorsal; V, ventral) (upper middle scheme). This eventually forms the gastrula with set body axes (upper right scheme). In the bottom part of the scheme, lack of *trim36* hampers cortical rotation due to disorganization of the microtubular array and results in ventralization of embryonic dorsal structures. (**B**) Representation of the dorsal mammalian neural tube at closure (left side) and the anencephaly defect as the result of lack of closure of the rostral cranial neuropore.

Most of the data available on the role of TRIM36 during development are obtained from the *Xenopus* model where the frog ortholog was found implicated in the definition of the very early developmental stages. The *Xenopus trim36* maternal transcript was found to be enriched at the vegetal cortex of oocytes where it was kept anchored by the RNA-binding protein dead-end 1 [20,25] (Figure 3A). The vegetal pole localization was also maintained after egg fertilization and up to 4- to 8-cell-stage embryos. Then, during early neurulation, *trim36* mRNA was detected outside the germ plasm in the developing neural tube, enriched at the midbrain-hindbrain boundary [20].

*Xenopus* embryos derived from fertilization of oocytes injected with antisense oligos against *trim36* develop normally until gastrulation but show delay in blastopore lip formation [20]. At the completion of gastrulation, the majority of them failed to form neural plates. By the early tailbud stages, *trim36*-depleted embryos were either radially ventralized, in the extreme cases, or formed partial axes lacking anterior structures in the less severe cases. *Trim36*-depleted embryos were shown to lack well-formed neural tubes, somites and notochords, in some cases forming unorganized lumps of somite tissue in the midline occupying the notochord left space. This defective dorsal tissue formation is possibly exerted along with β-catenin likely by acting upstream or in parallel to Wnt/β-catenin activation [20]. As detailed in the previous section, *trim36* is implicated in maintaining proper organization of the egg vegetal microtubular array that is necessary for cortical rotation to occur. In amphibian fertilized eggs, indeed, axial determination requires a dorsally directed rotational movement of the egg cortex, cortical rotation, at the first cell cycle, and which is driven by a parallel array of microtubules beneath the cortex, transporting dorsalizing factors to the dorsal side of the gastrula [26] (Figure 3A). Cortical rotation mediated by *trim36*-organized microtubule array is thus required to specify the dorso–ventral axis and induce dorsal structures and this action necessitates integral E3 ubiquitin ligase activity, although the molecular targets are still unknown [20] (Figure 3A).

A *Trim36* knock-out mouse line was generated and, unexpectedly, adult homozygous null mice were undistinguishable from heterozygous and wild-type mice. Further, no abnormal development and breeding of *Trim36* null mice were observed suggesting the presence of redundant genes in rodents [27]. However, when it comes to humans, *TRIM36* is another member of the TRIM subgroup I implicated in a birth defect. In fact, *TRIM18*/*MID1* is mutated in a developmental disorder characterized by midline defects, X-linked Opitz Syndrome (XLOS, OMIM 300000), and *TRIM1/MID2* is responsible for another X-linked form of intellectual disability (MRX101, OMIM 300928) [28,29]. A homozygous Pro508Thr missense mutation within the TRIM36 SPRY domain was detected in a 20-week-old fetus conceived of Indian consanguineous parents [19]. This fetus was affected by anencephaly (APH, OMIM 206500), an extreme form of neural tube defect that is quite common in India with 2.1:1000 births reported [30]. Anencephaly is characterized by the absence of cranial vault and brain tissues in the fetus and is incompatible with life. Early in embryogenesis, lack of closure of the cranium neuropore leads to neural tube defects leading to exencephaly at first and consequent degeneration of brain tissues in later stages [31] (Figure 3B). The mutation, detected in the first place by whole-exome sequencing, segregates with the disorder in the family [19]. To date, this remains the only *TRIM36* pathogenic variant so far reported in APH cases. Proline 508 is conserved in 26 species and the mutant protein is unstable if compared to the wild-type protein in HeLa cells and it is consistently less abundant in the APH fetus [19]. As observed for TRIM36 silencing, exogenous expression of the APH-associated mutation Pro508Thr leads to disorganized microtubules and apoptosis further supporting a pathogenetic role of this mutant [19].

Of note, TRIM36 is needed in both frog and human to determine the correct specification and closure during neurulation. Neural tube closure defects are observed also with ablation in frog of other 2 members of TRIM subgroup I, *trim18* and *trim1* [32]. The former is also responsible in humans with a genetic form of midline structure disorder, Opitz G/BBB Syndrome [28]. It is worth further investigating another unexpected common feature: both *Trim36* and *Trim18* mouse knock-out lines present with no or very mild phenotypes, suggesting the presence of different or compensatory mechanisms in mouse development [27,33].

## 5. TRIM36 Is Involved in Spermatogenesis

Spermatogenesis consists of three major events: the proliferation and differentiation of spermatogonia, the meiotic events of spermatocytes at prophase and morphological changes during differentiation from the haploid round spermatids to mature sperm. During haploid germ cell differentiation, referred to as spermiogenesis, molecules related to chromatin condensation, flagellum development and acrosome biogenesis are specifically expressed [18,34].

As mentioned above, the first papers reporting Trim36 describe its expression in haploid germ cells of mice testis. More specifically, Trim36 protein is detected in elongated spermatids, at the late steps of germ cell development, and its presence is restricted to the acrosomal region (Figure 2D) [16]. The acrosome is a large Golgi-derived vesicle located in the anterior head of the sperm, which develops during spermiogenesis and contains enzymes needed for the digestion of the zona pellucida of the egg [18,34]. Trim36 is not simply detected in the acrosome, but also participates in the acrosome reaction [16]. The acrosome is delimited by two membranes: the outer membrane, which underlies the plasma membrane, and the inner membrane, which overlies the nucleus. The acrosome reaction is a process that involves the fusion between the acrosomal membranes and the plasma membrane to expose and bind the plasma membrane of the egg through an exocytosis system triggered by increased Ca^2+^ levels [18,34,35]. When the mouse sperm was pre-treated with antibodies raised against Trim36, those raised against the N-terminal region, which contained RING domain but not the antibody against the C-terminal region, were able to inhibit the calcium-induced sperm acrosome reaction [16]. Beside the indication of Trim36 function during the acrosome reaction, this also suggests the need of Trim36 E3 ligase activity to exert this role. After the acrosome reaction, Trim36 is likely released as it is observed only in the acrosome of intact cells but not in the acrosome reacted cells [16]. Although the function of Trim36 in acrosome reaction is still unknown, it was speculated that SNARE-related components, e.g., Rab3a, SNARE and actin, might interact with it [16,18]. Moreover, whether Trim36 present in the crescent-shaped acrosome is somewhat associated with microtubules is still unknown. However, it is interesting to observe that another TRIM subgroup I member, TRIM9, is implicated in SNARE-regulated organelle fusion and that this can represent an additional common feature of this class of TRIM proteins [36].

Other reports further support the role of Trim36 in spermatogenesis. Trim36 was found upregulated in mouse spermatogonia cells in response to a reduction in Col1a1, a subunit of collagen type I, which suppresses self-renewal and accelerates differentiation [37]. Using buffalo as an experimental model, several studies were performed to analyze the effect of different media of germ lineage differentiation on Embryonic Stem (ES) cells differentiated into embryoid bodies. Both testicular cell–conditioned medium [38] and cumulus cell-conditioned media [39] induced buffalo ES cells into the germ lineage by increasing the expression of TRIM36 (at around 14 days). In addition, they showed that retinoic acid, directly involved in spermatogenesis developmental transitions [34,40], can anticipate the expression of TRIM36 early on culture (day 8) [41]. Further, differential proteomics and phosphoproteomics in buffalo, comparing pre-pubertal and pubertal testes, demonstrated that TRIM36 is upregulated with age suggesting a role in the spermatogenesis process in this species [42]. Furthermore, *Trim36* null mouse-derived spermatozoa showed lower total and progressive motility and less complex motility patterns [27]. However, time-series analysis of the occurrence of the acrosome reaction in *Trim36*-deficient spermatozoa showed no significant difference with respect to that of spermatozoa from wild-type mice [27]. As for other parameters, macroscopic and histological observation of testes and seminal vesicles of the *Trim36*-null mice did not show any significant differences in morphogenesis and spermatogenesis function. These mice had a normal number of epididymis-recovered spermatozoa and, most importantly, all males were fertile and produced normal litters [27]. However, when fertilization was carried on in vitro, *Trim36*-deficient spermatozoa were incapable of fertilizing oocytes [27]. The reason why *Trim36*-deficient sperms can fertilize in vivo but not in vitro is unknown. It is likely that in vivo additional factors, potentially components within the female tract, may compensate for the absence of active Trim36.

Whether human TRIM36 plays a role in spermatogenesis as well is still unknown. It is important to highlight that this process is different in rodents and humans and simple extrapolation of rodent insights to primates is not appropriate [43]. Probably the most supportive findings for an analogous role in human is provided in the marmoset, a New-World monkey, which shares a similar organization of spermatogenesis and testicular development as humans. In this model and similar to the other species analyzed to date, TRIM36 was detected in the acrosomal region of elongating and elongated spermatids following the initiation of adult spermiogenesis [44]. These data suggest that TRIM36 may also play such a role in human.

Thus, TRIM36 is expressed in the acrosomal region of different species and it has been reported to participate in the acrosome reaction and in spermatogenesis development, at least in vitro. However, the exact function in vivo and the molecular mechanisms involved still remain important open questions to address to fully understand TRIM36 physiological and pathological role in spermatogenesis.

## 6. Involvement of TRIM36 in Cancer

Likely connected to its role in affecting cell proliferation and apoptosis (see above), several reports associate variation of *TRIM36* expression levels or its methylation status with several types of cancers. Hypermethylation of *TRIM36* is detected in endometriosis-associated ovarian carcinoma and neuroblastoma tumors while its expression is significantly downregulated in several NSCLC cell lines, breast cancer and esophageal cancer. In prostate cancer, the results are less clear as both up- and down-regulation of TRIM36 have been reported. However, more recently, the majority of findings suggest a worse prognosis associated with reduced TRIM36 expression (see Table 1 and references therein). In one of these reports, TRIM36 was shown to be upregulated in prostate cancer patients and to delay the cell cycle of prostate cancer cells in vitro and in vivo [45]. Moreover, they show that TRIM36 can regulate the activity of the MAPK/ERK/MSK-1 pathway, a common altered pathway in tumorigenesis. The levels of activated phospho-MAPK/ERK and phospho-MSK1, and that of c-myc increased in TRIM36 knockdown cells (LNCaP cell line), while these levels decreased in the TRIM36-overexpressing PC3 cells [45]. These changes are associated with the increase of the G1/S phase markers Cyclin D1 and Cyclin E1 in TRIM36-silenced cells and an opposite trend in exogenously expressing TRIM36 [45].

## 7. TRIM36 Biochemical Roles

As we discussed above, TRIM36 is a microtubular protein acting in the organization of the cytoskeleton and through this function exerting roles in development and possibly in spermatogenesis and cancer. However, little is known about the signaling pathways in which TRIM36 is involved. Considering that the ERK1/2 signaling pathway mentioned above plays an important role in several developmental processes as well as during spermatogenesis, it is possible that the involvement of TRIM36 in this pathway can have important physiological and pathological implications.

The majority of TRIM family members act as E3 ubiquitin ligases within the ubiquitination cascade. Ubiquitination is a post-translational modification that consists of the covalent bond of Ubiquitin moiety(ies) usually on Lysine residues of the specific targets. This process is regulated through the intervention of a cascade of enzymes for: (i) the activation of the ubiquitin peptide (E1 ubiquitin activating enzyme); (ii) the conjugation of the activated ubiquitin (E2 ubiquitin conjugating enzyme); and (iii) the specific transfer of the properly oriented ubiquitin peptide(s) on the substrate (E3 ubiquitin ligase enzyme) [3]. The topologies and distribution of ubiquitin or ubiquitin chains on the targets via the action of this cascade of enzymes determine the fate of the target itself [54]. In many instances, TRIM-mediated ubiquitination of the substrate is meant for proteasome- or lysosome-mediated degradation but signals for the regulation of substrate activity or distribution can also be conveyed [54,55,56,57]. Not so many data are available for TRIM36 in this respect. Trim36 role in maintaining proper organization of the egg microtubular array requires intact E3 ubiquitin ligase activity [20] and the same was observed for the acrosome reaction [16]. In vitro ubiquitination assays using the *Xenopus trim36* demonstrated the ability to catalyze autoubiquitination [20] and it was shown in mammalian cells a preference to exert its E3 ligase activity in combination with the Ubc4 E2 conjugating enzyme [21]. To date however, no natural TRIM36 substrates have been reported. TRIM36 interacts with CENP-H [21] but it is not known if the latter is a TRIM36 ubiquitination substrate. It is possible that TRIM36 might be involved in controlling CENP-H degradation or CENP-H recruitment to the centromere, in a spatially and temporally dependent manner, thus impacting on cell proliferation and tumorigenesis.

## 8. Conclusions and Future Perspectives

We reviewed the currently available literature on TRIM36, an intriguing member of the TRIM family, which regulates microtubule organization and is involved in very different processes. Although several important findings are reported, there are still several questions left to be answered.

First, how TRIM36 regulates microtubule dynamics in such different compartments remains to be known. It is interesting to observe that any perturbations of TRIM36, silencing but also overexpression of the wild-type protein or of the anencephaly-associated mutant, cause microtubule disorganization. This would suggest a finely tuned control of its protein level, activity or availability. The experimental tools available now can allow to endogenously perturb TRIM36 in a much more controlled manner and in the relevant cell types. Further, we cannot exclude that some TRIM36 functions might be associated with movements or other activities over the microtubules and not necessarily due to an action on their organization/polymerization as a primary cause.

At developmental level, it would be interesting to analyze in more depth if TRIM36 expression observed in the neural tube both in *Xenopus* and mouse neurulation follow the same mechanisms and pathways and if the frog could represent a better model to study TRIM36-associated neural tube closure once the early xenopus-specific cortical rotation is bypassed. Further, the observed induction of TRIM36 upon retinoic acid during spermatogenesis can also underscore its ability to be regulated during early mammalian antero–posterior axis specification that is known to be regulated by retinoic acid gradients.

Finally, TRIM36 E3 ubiquitin ligase activity has been demonstrated in vitro but no natural substrates have been identified to date. We believe this is a crucial gap to fill to better understand the physiological and pathological pathways regulated by TRIM36 during development, spermatogenesis and cancer.

## Figures and Tables

**Table 1:** TRIM36 in different types of cancer.

Type of Cancer	TRIM36 Role	Ref.
Prostate cancer	TRIM36 mRNA expression is upregulated in patients’ tissues; no mutations gene detected.	[17]
	TRIM36 protein expression is negatively associated with patients’ poor prognosis. In the prostate cancer cell line PCa TRIM36 reduces cell growth. Smaller tumors generated in TRIM36 overexpressing PC3 cells mouse xenograft. TRIM36 is an androgen-responsive gene and enhances the efficacy of anti-androgen drugs in PCa cell lines.	[45]
	Increased *TRIM36* mRNA expression is associated with no PSA recurrence patients.	[46]
	Patients with low TRIM36 have poor progression-free survival. TRIM36 overexpression in LNCaP cells suppresses cell proliferation and migration and induces apoptosis by increasing Bax and TNFSF10.	[47]
Endometriosis	TRIM36 is hypermethylated in patients’ tissues.	[48]
Non-Small Cell Lung	TRIM36 mRNA is downregulated in NSCLC cell line.	[49]
Neuroblastoma	TRIM36 is hypermethylated in patients’ tissues.	[50]
Breast cancer	TRIM36 mRNA is downregulated in humanized breast and conventional subcutaneous mouse models.	[51]
Gastric cancer	TRIM36 mRNA expression is increased in patients receiving radiotherapy.	[52]
Esophageal cancer	Patients with low levels of TRIM36 (mRNA and protein) display larger tumor size, advanced stage, and lymph node metastasis.	[53]

## Data Availability

Not applicable.
